# Study protocol: effect of playful training on functional abilities of older adults - a randomized controlled trial

**DOI:** 10.1186/s12877-017-0416-5

**Published:** 2017-01-19

**Authors:** Jari Due Jessen, Henrik Hautop Lund

**Affiliations:** Center for Playware, Technical University of Denmark, Building 326, Kgs. Lyngby, 2800 Denmark

**Keywords:** Exergaming, Play, Functional abilities, Falls prevention

## Abstract

**Background:**

Loss of functional capabilities due to inactivity is one of the most common reasons for fall accidents, and it has been well established that loss of capabilities can be effectively reduced by physical activity. Pilot studies indicate a possible improvement in functional abilities of community dwelling elderly as a result of short-term playing with an exergame system in the form of interactive modular tiles. Such playful training may be motivational to perform and viewed by the subjects to offer life-fulfilling quality, while providing improvement in physical abilities, e.g. related to prevent fall accidents. The RCT will test for a variety of health parameters of community-dwelling elderly playing on interactive modular tiles.

**Methods:**

The study will be a single blinded, randomized controlled trial with 60 community-dwelling adults 70+ years. The trial will consist an intervention group of 30 participants training with the interactive modular tiles, and a control group of 30 participants that will receive the usual care provided to non-patient elderly. The intervention period will be 12 weeks. The intervention group will perform group training (4–5 individuals for 1 h training session with each participant receiving 13 min training) on the interactive tiles twice a week. Follow-up tests include 6-min Walk Test (6MWT), the 8-ft Timed Up & Go Test (TUG), and the Chair-Stand Test (CS) from the Senior Fitness Test, along with balancing tests (static test on Wii Board and Line Walk test). Secondary outcomes related to adherence, motivation and acceptability will be investigated through semi-structured interviews. Data will be collected from pre- and post-tests. Data will be analyzed for statistically significant differences by checking that there is a Gaussian distribution and then using paired *t*-test, otherwise using Wilcoxon signed-rank test. “Intention to treat” analysis will be done.

**Discussion:**

The trial tests for increased mobility, agility, balancing and general fitness of community-dwelling elderly as a result of playing, in this case on modular interactive tiles. A positive outcome may help preventing loss of functional capabilities due to inactivity.

**Trial registration:**

ClinicalTrials.gov: Nr. NCT02496702, Initial Release date 7/7–2015.

## Background

This protocol works with three main concepts:

- Playware: defined as “Intelligent hard- and software that creates playful experiences for people of all ages.” [1].
- Exergames: games that require the user to be physically active to play the game, thus the games “…incorporate technology, play and physical activity…” [2]. These are a subset of playware.
- Interactive modular tiles (IMT): The product used in this project, which are developed at Center for Playware and are used to create exergaming and play [3].

These concepts are used with elderly citizens in interventions, which should be playful and motivational to perform, in order to prevent loss of functional capabilities and related falls.

Fall accidents among elderly inside or outside of their homes is the most common cause of fractures and hospitalization. Falling has many human and economic costs and within health prevention training of elderly in order to prevent falls is an important issue. Elderly who are very sedentary have an increased risk of falling. One third of senior citizens age 65–80 fall at least once a year [4], while it is half of the population over 80 [5]. Hospitalizations associated with falls in Denmark account for about 13,000 per year in 2005 and are expected to rise to almost 24,000 per year in 2030. 10–20% of the falls result in serious injury, about 5% results in fractures and 1% are hip fractures. Of the people falling 20% die within a year after the incident [5–7].

Loss of functional capabilities due to inactivity is one of the most common reasons for falling, and it has been well established that loss of capabilities can be effectively reduced by physical activity [8, 9]. In order to assess functional capabilities Rikli and Jones [10] developed the senior fitness test to asses fitness parameters in older adults. They further defined functional fitness as “having the physiological capacity to perform normal everyday activities safely and independently without undue fatigue.” [9].

The Senior Fitness Test [10] scores have shown to be highly correlated with loss of functional abilities, as such keeping older adults physical active is the goal of this study. Investigating the functional capabilities of participants can give insights to how the IMT can help elderly avoid fall related injuries. Further, research has shown that barriers for elderly being physically active include poor health, fear of injury or lack of motivation, opportunities or companionship. Exergames have the potential to diminish some of these barriers.

There has been performed little research of this kind in the area of exergames as a tool to perform such exercises [2, 8].

A review and meta-analyse of exergames for older adults area found eighteen studies [8], an earlier review done at the point of planning this intervention found seven studies [2]. The reviews conclude that exergames have an overall positive effect on improving balance and mobility, but they also point to that there is a need for more studies with a higher degree of methodological quality (less risk of bias) [2, 8].

The goal of this project is to investigate and validate the use of one type of exergaming training tool the MOTO tiles to prevent loss of function abilities and decrease chance of falls among elderly people. The project will compare training with the MOTO tiles to usual care in the field with the aim of investigating whether the training will result in an improved physical abilities compared to usual care.

A pilot study of the use of the MOTO tiles in a municipality with no control group was conducted in 2012. This initial pilot study showed that the use of playware [3] technology for training and play could provide statistically significant progress in physical abilities [11]. The pilot study had 16 participants and significant improvement in the balance (Line walk – LW) (65%), endurance (6 min walking test - 6MWT) (26%), strength in the lower body (chair stand - CS) (20%) and dynamic balance and agility (Timed Up and Go - TUG) (18%) after just ten training times of 12–15 min per session [11]. In addition to the physical benefits, it was highlighted in subsequent interviews that it was fun to work out on the tiles, and 80% wanted to continue with this kind of playful training [7].

The indications from this pilot study and a MAST [12] investigation of the MOTO tiles have indicated the need for a RCT study to validate both the technology and play as a motivation for exercises among elderly to prevent loss of functionality and falls.

The MOTO tiles is a distributed system consisting of digital tiles that are able to sense pressure and light up in a rainbow of different colors. The tiles are designed as puzzle pieces and can be assembled and disassembled in a matter of minutes.

Detecting pressure through the pressure sensor and the many different colors the tiles can light up in, a variety of different games have been created where the player have to move around and step on the different tiles [3, 10].

## Methods/Design

### Objectives

The research hypothesis is that playful training here in the form of the IMT, will make participant perform better on the follow-up tests (6-min Walk Test (6MWT), the 8-ft Timed Up & Go Test (TUG), and the Chair-Stand Test (CS) from the Senior Fitness Test [10], than participants not using the IMT. Further it is expected that the participant will perform better on balancing tests (static test on Wii Board and Line Walk test), have a high degree of adherence (participation in +90% of the training), express that IMT is motivating for the training and IMT have a high acceptability.

### Trial design

This study will investigate the use of one form of exergames called IMT, and how this compares to usual care of elderly people 70+. The trial will consist of two groups, one for training with the IMT and one group for usual care that will receive the care provided to non-patients elderly, which at this moment is no additional treatment other than recommendations.

The study will be a single blinded, randomized controlled trial. The study will be funded by the patient@home project, and equipment by Entertainment Robotics. Concealed allocation and intention-to-treat analysis will be used. Measurements will be taken at baseline and after intervention. Upon acceptance the protocol will be registered in clinicaltrials.gov.

Potential participants will be interviewed either by the primary investigator or student assistants at Center for Playware, and assessed for inclusion and exclusion criteria. After interviews the primary investigator will enroll participants. After pre-testing, the participants will be randomized and allocated to either control or intervention group (ratio 1:1) by an individual who is not involved in any of the other parts of the study. The randomization sequence will be created using the online platform www.randomization.com. Allocation will be done after pre-testing, as we have earlier experienced that some potential participants dropped out of the study just before starting.

The pre- and post-test will be done by an external physiotherapist blinded to the participants group allocation. The blinding will be done by using participation numbers and pre- and post-data will be obtained on different data-sheets. Participants, supervisors and care givers will not be blinded.

The trial’s results will be reported using domains and categories described in the taxonomy developed by the Prevention of Falls Network Europe, to allow future synthesis of evidence, or study replication [13].

### Study settings

In Denmark, care for the elderly by the municipalities consists of many different services. Care is given in form of nursing homes, day centers and care at the private homes depending on the elderly and their abilities. The care is granted by the municipalities, and is an ongoing negotiation on the amount of money for elderly care and other services provided by the municipalities.

Activities for elderly consist of volunteer activities such as gymnastics (local teams often with public support), day centers where elderly is screened and appointed to, or rehabilitation normally after operations or hospitalization. In a recent study [11], elderly at a day center participated in exercising with playware technology.

In this study, the participants will be participating in training at facilities appointed by the participating municipalities. The intervention will consist of 2 weekly group training sessions for the participants.

The interventions group will train using interactive modular tiles. The tiles include preprogrammed games that create playful training for the participants. The tiles are described in Lund [3], and Lund and Jessen [11]. The tiles are approx. 30 × 30 cm and can light up in a variety of colors and detect pressure. The rules of the games developed for the tiles vary, but in general the player should press the tiles that light up. Another tile will then light up getting the player to move from tile to tile in order to play the game. The player controls the speed and intensity of the games by their choice of movement speed, if they move quicker the game will become quicker. The games are focused on the lower body (primary legs).

The control group will receive “usual care”, which here refers to normal day activities.

**Fig. 1:**
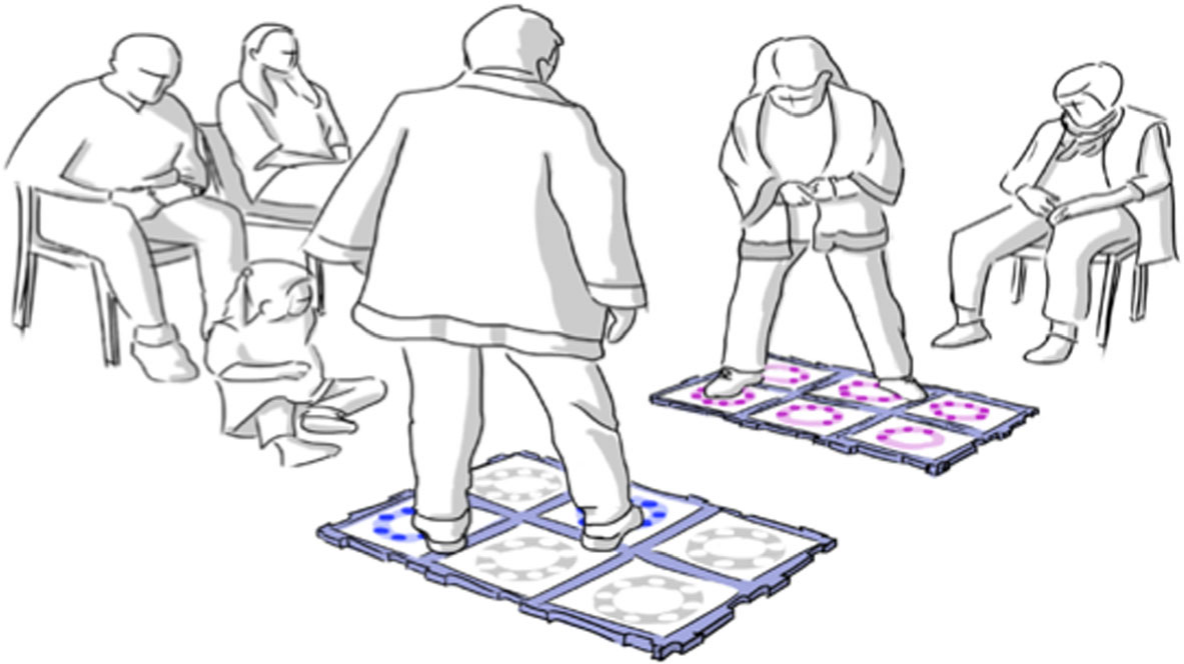
Group training with the IMT

The intervention is done in the form of groups of 4–5 participants per set of tiles, with 2–3 sets of tiles at a time (see Fig. 1: Group training with the IMT). As more sets can be used it is possible to make groups of more people. The training will consist of 1.5–3 min of training (depending on the game) on tiles and then rest while the other 2–3 participants train (4–6 min of break). Then the participants will train for 1.5–3 min again until each participant has received a total of 13 min of training. The control group will not train.

### Inclusion criteria

Community-dwelling men and women aged 70 and above will be included in the project. As the project is a preventive study there is no special requirements to the participants other than the above.

### Exclusion criteria

Participants will be excluded if any of the following are identified:

- A previous diagnosis of strong dementia or a cognitive decline that prevents the understanding of simple instructions or guidelines;
- A previous stroke with a severe neurological impairment, such as loss of strength, and perceptual or language limitations;
- A severe visual deficiency;
- Inability to maintain a standing position, even with the use of a walking aid or other device;
- Participating in rehabilitative training.

### Primary outcome

The primary outcome measures using pre- and posttests of the TUG, 6MWT and CS. The tests 6MWT, TUG and CS are described in the Senior Fitness Tests Manual [10]. The Senior Fitness Test [10] score fitness standards (performance cut points) that are associated with older adults ability to function independently. More than 2000 older adults have been scored, and the cut points’ accuracy and consistency have been validated as clear predictors of physical independence. The test can classify each individual into one of four levels (above average, normal range, below average or low functioning) and can be used to assess the individual’s health risk level.

Three different tests from the Senior Fitness Test [10] are focusing on health risk prevention, such as mobility, agility, balancing and general fitness, and are well established for testing a variety of health parameters in community-dwelling elderly. These tests include the 6MWT, the 8-ft TUG and the CS.

Studies have shown that the 6MWT can be used as a fall risk indicator specifically for frail elderly [14]. Furthermore, the 6MWT not only measures aerobic fitness and mobility, but also incorporates components of leg strength, balance, reaction time and vision.

It has been shown that the TUG reflects a combination of sensory, motor and strength abilities [15] and can be used as a measurement of functional mobility [16]. It has also been shown that the TUG can be a tool for discriminating between future fallers and nonfallers [14]. It has been demonstrated that the 8-ft version of TUG have similar qualities for agility and dynamic balancing and that this test is a reliable test for predicting future fallers and nonfallers from among the community-dwelling elderly population [17]. The CS measures lower body strength and endurance and the CS has been shown that the CS is a reliable and valid indicator of lower body strength in generally active, community-dwelling older adults [18].

### Secondary outcomes

The following secondary outcomes will also be collected as part of the intervention:

- Line Walk test which is a test to measure balance.
- Static Balance will be measured using Wii Balance Board, which have proven to be a valid measure of balance [19]. This measure will be done using an application developed by Francesco Sgró and colleagues [19].
- Adherence to training
- Motivation for training
- Acceptability of IMT

Adherence to the training will be measured by registering the number of times the participants participate and how much they participate at each session. Semi-structured interviews will be done with the participant after the intervention to investigate motivation and acceptability of the IMT.

### Sample size/power analysis

The data from the pilot study (described in Lund and Jessen [11]) was used to do a power calculation. The results showed that for a power of 80 there is a need for 20 persons in each group. As the population of community-dwelling elderly is expected to exhibit a high variance in functional abilities (higher than the population of the pilot study) we have increased the number of participants to 30 in each group. This also allows for dropouts from the study, which we experienced before initializing the pilot study.

### Statistical methods

#### Primary outcome analysis

Data will be collected from pre- and post-tests. Data will be analyzed for statistically significant differences by first checking that there is a Gaussian distribution and the using paired *t*-test, otherwise using Wilcoxon signed-rank test. The Wilcoxon Signed-Rank Test is selected because the population of community-dwelling elderly is expected to exhibit a high variance in functional abilities. Therefore a normal distribution of the population’s test scores cannot be assumed. The Wilcoxon Signed-Rank Test is a statistical hypothesis test suitable under such circumstances.

“Intention to treat” analysis will be done. These two conditions (ie, all participants, as randomized) are widely recommended as the preferred analysis strategy.

#### Secondary outcome analysis

Data from the LW test will be calculated in the same way as the primary outcomes.

Adherence will be calculated for the average and standard deviation for the participant. This will be compared to a goal of minimum 90% adherence to training.

Motivation will be based on the average scores and standard deviation from the participant interviews (questionnaire on motivation) as will the acceptability.

There are no other analysis planned for the study.

## Discussion

Investigating if short-term playful training on the IMT will increase mobility, agility, balancing and general fitness of community-dwelling elderly is the goal of the study. A positive result may help creating more training opportunities to help prevent loss of functional capabilities due to inactivity.

The study will bring further insights to if exergames, a field under development with only few RCTs still done, can have a positive effect on the community-dwelling older adults. Further, the IMT will be assessed to see if the way of creating playful experiences as they do is usable in a larger study.

The study has several logistic challenges, as the participants are living at home but most have balancing problems, which prevent them from getting to a training facility on their own. This have been sought dealt with by going into a cooperation with two Danish care-centers, where elderly come one or two times a week to do different activities (arts and social activities mainly). This means that it could be argued that the participants of the study is only a subset of the elderly in general, but this subset is fairly representative for the municipality.
